# Plasma and CSF pharmacokinetics of meropenem in neonates and young infants: results from the NeoMero studies

**DOI:** 10.1093/jac/dky128

**Published:** 2018-04-19

**Authors:** Eva Germovsek, Irja Lutsar, Karin Kipper, Mats O Karlsson, Tim Planche, Corine Chazallon, Laurence Meyer, Ursula M T Trafojer, Tuuli Metsvaht, Isabelle Fournier, Mike Sharland, Paul Heath, Joseph F Standing, NeoMero Consortium, Cinzia Auriti, Susanna Esposito, Pugini Lorenza, Mari-Liis Ilmoja, Nijole Drazdiene, Kosmas Sarafidis, Georgios Mitsiakos, Michiel van der Flier, Paul Clarke, Andrew Collinson, Samir Gupta, Mark Anthony, Mark Thomas, Santosh Pattnayak, Jonathan Davis, Heike Rabe, Elizabeth Pilling, Srini Bandi, Ajay Sinha

**Affiliations:** 1 Department of Infection, Inflammation and Rheumatology, Great Ormond Street Institute of Child Health, University College London, London, UK; 2 Department of Pharmaceutical Biosciences, Uppsala University, Uppsala, Sweden; 3 Department of Microbiology, University of Tartu, Tartu, Estonia; 4 Institute for Infection and Immunity, St George’s, University of London, Cranmer Terrace, London, UK; 5 INSERM SC10-US019, Villejuif, France; 6 Neonatal Intensive Care Unit, Department for Women and Child Health, University of Padua, Padua, Italy; 7 Tartu University Hospital, Tartu, Estonia

## Abstract

**Background:**

Sepsis and bacterial meningitis are major causes of mortality and morbidity in neonates and infants. Meropenem, a broad-spectrum antibiotic, is not licensed for use in neonates and infants below 3 months of age and sufficient information on its plasma and CSF disposition and dosing in neonates and infants is lacking.

**Objectives:**

To determine plasma and CSF pharmacokinetics of meropenem in neonates and young infants and the link between pharmacokinetics and clinical outcomes in babies with late-onset sepsis (LOS).

**Methods:**

Data were collected in two recently conducted studies, i.e. NeoMero-1 (neonatal LOS) and NeoMero-2 (neonatal meningitis). Optimally timed plasma samples (*n* = 401) from 167 patients and opportunistic CSF samples (*n* = 78) from 56 patients were analysed.

**Results:**

A one-compartment model with allometric scaling and fixed maturation gave adequate fit to both plasma and CSF data; the CL and volume (standardized to 70 kg) were 16.7 (95% CI 14.7, 18.9) L/h and 38.6 (95% CI 34.9, 43.4) L, respectively. CSF penetration was low (8%), but rose with increasing CSF protein, with 40% penetration predicted at a protein concentration of 6 g/L. Increased infusion time improved plasma target attainment, but lowered CSF concentrations. For 24 patients with culture-proven Gram-negative LOS, pharmacodynamic target attainment was similar regardless of the test-of-cure visit outcome.

**Conclusions:**

Simulations showed that longer infusions increase plasma PTA but decrease CSF PTA. CSF penetration is worsened with long infusions so increasing dose frequency to achieve therapeutic targets should be considered.

## Introduction

Sepsis and bacterial meningitis are major causes of mortality and morbidity in neonates and infants and can be considered as part of a single continuum in that septic infants can develop meningitis.^1^^,^^2^ Late-onset sepsis (LOS) is defined as sepsis starting 72 h or more after birth.

Meropenem is a broad-spectrum carbapenem antibiotic with activity against common pathogens causing neonatal sepsis and meningitis. It is bactericidal against *Escherichia coli, Klebsiella* spp., *Enterobacter* spp. and *Pseudomona*s spp.,^3^ which are known to cause LOS, and also against pathogens responsible for bacterial meningitis such as *Streptococcus agalactiae* (i.e. group B streptococci), *E. coli, Listeria monocytogenes, Haemophilus influenzae, Streptococcus pneumoniae* and *Neisseria meningitidis*.^2^^,^^4^

Meropenem pharmacodynamics (PD) is usually described by the percentage of time when concentrations are above the MIC (%*T*_>MIC_). Approximately 40%*T*_>MIC_ is believed to be sufficient for a bactericidal effect;^5^^,^^6^ however, for immunocompromised patients, including neonates, higher targets have been suggested^7^ and a recent study looking at meropenem PD indices determined the PD target for neonates to be 61%*T*_>MIC_.^8^ For lower respiratory tract infections, %*T* >* *5× MIC was suggested.^9^ Meropenem is predominantly renally eliminated and approximately 75% is excreted unchanged in the urine.^10^^,^^11^ As a polar, hydrophilic molecule, meropenem’s penetration into the CSF through the blood–brain barrier is limited under normal, healthy conditions, but this may increase when the meninges are inflamed.^12^^,^^13^

Meropenem is currently unlicensed in infants below 3 months of age and, whilst there are studies describing its pharmacokinetics (PK) in neonates and infants,^14–18^ only Smith *et al.*^15^ studied both plasma and CSF concentrations, including single CSF concentrations from only six patients. Since the concentration–time profile of meropenem differs between plasma and CSF, taking the ratio of CSF to plasma is confounded by time after dose. To correctly assess the fractional CSF penetration necessitates sufficient sample numbers and timing for a model-based estimation. Consequently, sufficient information on meropenem’s disposition to infer dosing in this population is lacking. The NeoMero Consortium recently completed two studies with PK sampling from both plasma and CSF: NeoMero-1 [‘Efficacy, pharmacokinetics and safety of meropenem in subjects below 90 days of age (inclusive) with clinical or confirmed late-onset sepsis: a European multicentre randomised phase III trial’] compared meropenem with standard of care (β-lactam plus aminoglycoside);^19^ and NeoMero-2 [‘Pharmacokinetics and safety of meropenem in subjects below 90 days of age (inclusive) with probable and confirmed meningitis: a European multicentre phase I-II trial’]. Since some patients recruited to NeoMero-1 developed meningitis and transferred to NeoMero-2, the aim of this study was to report a joint analysis of the plasma and CSF PK results from both studies. We further sought links with outcome (PD) in NeoMero-1 (LOS) in order to inform dosing for LOS.

## Patients and methods

### Ethics

In NeoMero-1, subjects were recruited from 15 centres in six different countries (Estonia, Greece, Italy, Lithuania, Spain and Turkey). There were, on average, 8 subjects/centre (range 1–25). In NeoMero-2, there were 21 centres in seven countries (Estonia, Greece, Italy, Lithuania, the Netherlands, Spain and the UK) recruiting, on average, 2 subjects/centre (range 1–9). Independent Ethics Committees in each country approved the studies, which were registered on EudraCT (2011-001515-31 and 2011-001521-25) and clinicaltrials.gov (NCT01551394 and NCT01554124).

### Patient recruitment

The inclusion criteria for NeoMero-1 were: diagnosis of sepsis and postnatal age (PNA) ≤90 days, and ≥72 h of life at sepsis onset. Sepsis was defined as: (i) sepsis confirmed by a positive bacterial culture, accompanied by an abnormal clinical or laboratory measurement; or (ii) clinical sepsis, i.e. in case the bacterial culture was negative, clinical and laboratory criteria defined by either EMA^20^ or Goldstein^21^ had to be met, depending on the postmenstrual age of the infant. An infant could not be included in the trial if they received systemic antimicrobials more than 24 h prior to randomization. The inclusion criteria for NeoMero-2 were: PNA ≤90 days, clinical signs indicating bacterial meningitis, or pleocytosis, or a positive Gram stain from the CSF. An infant was not included in the trial if a CSF device was present or if meningitis was proven to be non-bacterial. In both studies, written informed consent was required from the parents or legal guardians of the infant. Exclusion criteria in both studies were: presence of renal failure, severe congenital malformations, causative pathogen resistant to meropenem (known or suspected) and known intolerance or contraindications to meropenem treatment.

### Meropenem administration

Meropenem was supplied by Chiesi Pharmaceuticals, Italy. A 12 hourly dose interval was used in patients with <32 weeks’ gestational age (GA) and <2 weeks’ PNA, and 8 hourly in all others. For LOS 20 mg/kg was used and for meningitis 40 mg/kg was used, these doses and frequencies having been previously used off-label. Meropenem was infused over 30 min, which was determined using a now-published model^15^ (interim version provided during the protocol development by Professor Caparelli) and simulating the PTA with different infusion lengths, specifically bolus, 30 min, 1 h and 2 h infusions. Whilst the 2 h infusion gave a greater PTA, concerns about use of line space and possible reduced CNS penetration led to a pragmatic decision to use 30 min infusions.

### PK sampling

Plasma sampling for PK was designed to minimize invasiveness whilst maximizing information gained. Three optimally timed PK samples were therefore decided to be taken from ≥56 subjects, spread evenly over each of the following age groups: <32 weeks GA and <2 weeks PNA; <32 weeks GA and ≥2 weeks PNA; ≥32 weeks GA and <2 weeks PNA; and ≥32 weeks GA and ≥2 weeks PNA. The remaining patients would contribute a single trough sample. Owing to logistical constraints in that meropenem could be started at any time, and in an emergency setting, PK sampling was planned to take place at steady-state. The PK model mentioned in the previous section and demographics from a previous European neonatal/infant sepsis study^22^ were used to define optimal sampling times. ED-optimal design (PopED software^23^) was used, including interindividual variability. For the three-sample cohort, optimal times were immediately following the end of the infusion, 5–6 h post-dose for 8 hourly dosing, 7–8 h post-dose for the young preterm group dosed 12 hourly and a trough sample immediately before a dose. For the single sampling optimal design, a trough sample proved most informative. Since it was not possible to take CSF samples specifically for PK, these were opportunistically collected when lumbar puncture was performed for other study-specific purposes, with accurate recordings of time, date and dose history.

### Sample handling and meropenem assay

Blood samples were immediately spun down and plasma extracted. All samples were frozen within 1 h of collection and stored at −80°C prior to analysis. Ultra-HPLC coupled with tandem MS (UHPLC-MS/MS) was used to determine plasma and CSF meropenem concentrations. To prepare 50 μL of plasma and CSF samples for the analysis, protein precipitation with methanol and microfiltration (using 0.22 μm filters), respectively, were used. In both cases we used ertapenem as an internal standard. The limit of detection for the plasma assay was 10 ng/mL and the limit of detection for the CSF assay was 2 ng/mL. The between-day variability was 4.1%–5% over the whole calibration range, including the limit of quantification (LoQ) of 80 ng/mL for the plasma assay; and 3%–5% for the CSF assay with an LoQ of 6 ng/mL.

### PK modelling

PK modelling was undertaken using the first-order conditional estimation method with interaction (FOCEI) in NONMEM 7.3 (ICON Development Solutions, Ellicott City, MD, USA).^24^ Firstly the plasma model was determined, followed by addition of a compartment for the CSF concentrations. For the plasma PK, one-, two- and three-compartment structural models were tested to define the basic structural model. Between-subject variability was assumed to follow a log-normal distribution, and for the residual error, proportional, additive, combined and Box–Cox power transformation (with both the shape and the scedasticity parameters estimated^25^) were tested. To delineate size and age from other possible covariates, body weight and postmenstrual age were included *a priori* in the model with allometric weight scaling and a function describing renal function maturation, respectively.^26^ The parameters of the maturation function were fixed to values from a previous study of human glomerular filtration development.^27^ However, since meropenem is renally cleared, and renal function improves in the days after birth independently of postmenstrual age, we also tested the effects of PNA and serum creatinine (corrected for postmenstrual age^28^) on CL. A covariate was included in the final model, if (after the inclusion) it produced a drop in the objective function value (OFV) (ΔOFV) of >6.63, which corresponds to a *P* value of <0.01. The significance of a covariate was also tested by a randomization test^29^^,^^30^ (*n *=* *1000) performed using PsN.^31^ Since most patients were expected to contribute one CSF sample, the CSF volume was fixed to 0.15 L/70 kg^32^ and so two parameters were estimated: the inter-compartmental CL between plasma and CSF, and the fraction of meropenem penetration from the central compartment into the CSF. Markers of CNS inflammation (CSF proteins, lactate concentration, glucose concentration or number of white blood cells per unit of volume) may correlate with blood–brain barrier function; therefore, the effects of these covariates on penetration fraction were investigated. When these covariates were missing, they were replaced with the median.

Basic goodness-of-fit plots [observations versus predictions, conditional weighted residuals (CWRES) versus time and prediction] in addition to visual predictive checks with 1000 replicates were used during model-building and to select the final model. A non-parametric bootstrap analysis was performed (*n *=* *1000) on the final model to test parameter robustness and derive uncertainty around the parameter estimates.

### PD analysis of LOS

For NeoMero-1 patients with culture-proven Gram-negative bloodstream infections for which an MIC could be determined and who received at least 24 h of treatment, the model was used to generate individual AUC_0–24_:MIC, *C*_max_:MIC, *C*_min_:MIC and %*T*_>MIC_. These were compared with whether the patient successfully completed the treatment course with clinical/laboratory improvement (success) or if treatment had to be modified at the discretion of the treating physician or the patient died (failure). These endpoints were measured at the test-of-cure visit 2 ± 1 days after the end of planned therapy (11 ± 3 days). The Kruskal–Wallis test was used to compare the two groups.

### Simulations

Monte Carlo simulations (*n *=* *1000) using the final model estimates were used to generate %*T*_>MIC_ curves for different dosing regimens and the following MIC values: 0.25, 0.5, 1, 2, 4, 8 and 16 mg/L. The unbound fraction of meropenem was fixed to 0.98.^33^ The %*T*_>MIC_ curves were generated using plasma (for LOS) or CSF (for meningitis) predictions. The PD target was set to 61%*T*_>MIC_;^8^ and simulations were done for all four age groups.

## Results

### Demographics and PK samples

A total of 167 patients underwent PK sampling in the NeoMero studies, with 123 from NeoMero-1 (5 of whom were diagnosed with probable or confirmed bacterial meningitis and transferred to NeoMero-2) and 49 (including the 5 from NeoMero-1) in NeoMero-2. At enrolment, their median (range) weight was 2.12 (0.48–6.32) kg, PNA was 13 (1–90) days and gestational age (GA) was 33.3 (22.6–41.9) weeks. Patients from the NeoMero-1 study were more premature (median GA of 31.9 weeks versus 37.1 weeks in NeoMero-2) and therefore also weighed less than the patients from the NeoMero-2 study. Demographics are presented in Table 1.

**Table 1.** Demographics of included subjects

	All data	NeoMero-1	NeoMero-2
Number of subjects^a^	167	123	49
Weight (kg), median (range)	2.12 (0.48–6.32)	1.68 (0.48–5.01)	3.11 (0.60–6.32)
GA (weeks), median (range)	33.3 (22.6–41.9)	31.9 (22.6–41.3)	37.1 (23.4–41.9)
PNA (days), median (range)	13 (1–90)	15 (3–83)	9 (1–90)
Postmenstrual age (weeks), median (range)	37.4 (23.7–51.3)	36.0 (23.7–51.3)	38.8 (24.9–51.1)
Female, *n* (%)	78 (46.7)	59 (48.4)	19 (42.2)
Number of plasma samples	401	255	147
Plasma samples per patient, mean	2.4	2.1	3.0
Number of CSF samples	78	32	46
CSF samples per patient, mean	0.47	0.26	0.94
Plasma concentration (mg/L), median (range)	7.94 (0.01–147.7)	5.27 (0.01–147.7)	12.4 (0.1–139.0)
CSF concentration (mg/L), median (range)	1.58 (0.04–35.4)	1.23 (0.04–7.34)	1.90 (0.05–35.4)
Plasma time after the dose (h), median (range)	5.66 (0–12.4)	5.93 (0–12.4)	5.19 (0–12.2)
CSF time after the dose (h), median (range)	5.27 (0–12.0)	5.99 (0–12.0)	5.03 (0–11.5)
Creatinine (μmol/L), median (range)	32.0 (3.54–197.4)	34.5 (3.54–197.4)	27.0 (6.0–133)
C-reactive protein (mg/L), median (range)	23.0 (0.3–280)	23.2 (0.3–242)	22.4 (0.4–280)
Procalcitonin (ng/mL), median (range)	2.7 (0.1–377.2)	2.8 (0.1–128.6)	1.8 (0.1–377.2)

For creatinine, C-reactive protein and procalcitonin, the summary statistics represent all samples recorded during the study.

Day 0 = first day of life.

Weight, PNA and GA–at enrolment.

a Five infants switched from NeoMero-1 to NeoMero-2.

**Figure 1.**
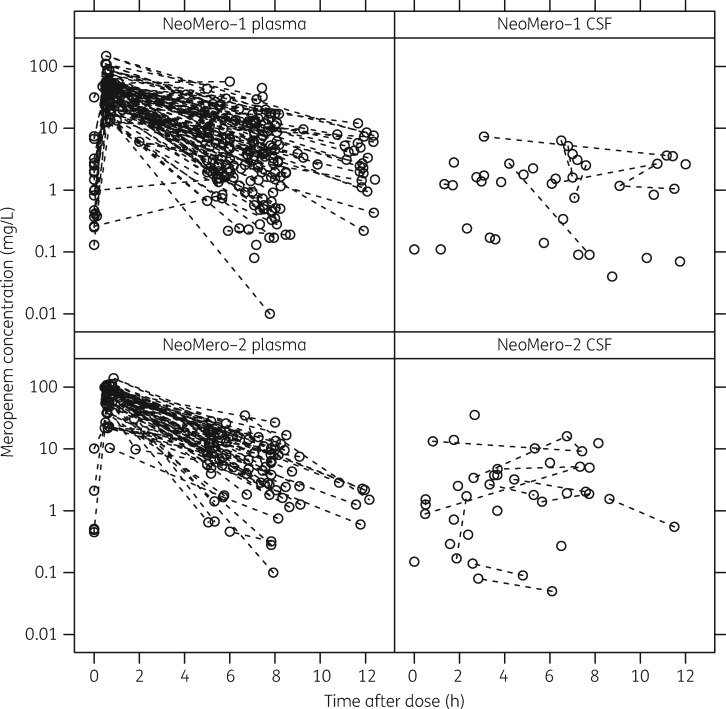
Plot of meropenem concentration versus time after dose for plasma and CSF. The top two plots show data for NeoMero-1 and the bottom two plots show data for NeoMero-2. Data points from the same individual are joined with a broken line (these are not always taken from the same dosing interval).

Three optimally timed plasma samples were collected from 109 patients, whilst 44 provided a single trough sample. Sampling numbers from the remaining patients were two (nine patients), four (four patients) and seven (one patient). There was an even spread in PNA and GA of optimally timed samples with 25, 18, 20 and 24 patients, respectively, providing three optimal samples in each of the pre-defined age categories (<32 weeks GA and <2 weeks PNA; <32 weeks GA and ≥2 weeks PNA; ≥32 weeks GA and <2 weeks PNA; and ≥32 weeks GA and ≥2 weeks PNA). Following sample analysis, 11 meropenem peak plasma samples were below 10 mg/L indicating possible data entry error and were thus excluded from the analysis to prevent biasing the model development. The influence of these data points was tested with the final plasma model and, although the changes in the typical final model parameter estimates were below 10%, the interindividual variability and the uncertainty approximately doubled, again giving a reason for their exclusion. The data set for model-building therefore contained 401 plasma samples and 78 CSF samples (CSF was obtained from 56 patients). The median (range) of CSF sampling time was 5.27 (0–12.0) h post-dose. Plots of the raw data are presented in Figure 1. One CSF protein concentration was also excluded from the analysis, as it was not deemed biologically plausible (102 g/L).

### Meropenem clearance is significantly related to renal function, and CSF penetration to CSF protein concentration

The final plasma population PK model was a one-compartment model. Weight was included with allometric scaling (with exponents fixed to 1 for central volume and 0.632 for CL^27^) and postmenstrual age was included with a maturation function (with parameters fixed to values from a study of renal development^27^). PNA did not significantly improve the model fit; however, serum creatinine concentration (standardized by postmenstrual age) proved to have a significant effect on meropenem clearance (ΔOFV of 19.7) and was therefore also included in the final model.

An additional compartment was added to describe meropenem CSF PK and to estimate the penetration of meropenem from plasma to the CSF. Out of the tested covariates, both the CSF lactate concentration and the CSF total protein concentration proved to significantly explain the CSF penetration (ΔOFVs of 24.3 and 24.4, respectively). However, since there were fewer missing measurements for CSF protein concentration (there were 86 protein and 54 lactate concentrations available, with 58 and 41 of these, respectively, taken at the time of CSF meropenem sampling), this covariate was included in the final model. The significance of the covariates was also confirmed by a randomization test.^29^^,^^30^

Initially, a proportional model was chosen for the residual error of both plasma and CSF data; however, Box–Cox power transformations of the residual error^25^ resulted in an improved fit (ΔOFV of 67.8) and there was an improvement in the distribution of the residuals; therefore, this residual error model was used. The estimate for the scedasticity parameter corresponding to the CSF concentrations was approximately zero and there was no improvement whether it was estimated or not; therefore, it was fixed to zero. The final model parameters are presented in Table 2.

**Table 2.** Population PK model final parameter estimates

	Mean	SE	%CV	η-shrinkage (%)	Bootstrap, median (95% CI)
CL (L/h/70 kg)	16.7	1.07	—	—	16.7 (14.7, 18.9)
θ_creatinine	−0.40	0.094	—	—	−0.40 (−0.58, −0.21)
*V* (L/70 kg)	38.6	2.15	—	—	38.6 (34.9, 43.4)
CL_CSF_ (L/h/70 kg)	0.017	0.004	—	—	0.016 (0.001, 0.030)
CSF uptake^a^	2.39	0.205	—	—	2.38 (2.01, 2.82)
θ_CSF proteins^a^	−0.17	0.110	—	—	−0.17 (−0.43, 0.015)
IIV on CL	0.255	0.058	50.5	13.5	0.248 (0.154, 0.370)
IIV on *V*	0.153	0.059	39.1	31.0	0.154 (0.042, 0.282)
Cov IIV CL-*V*	0.167	0.055	—	—	0.163 (0.070, 0.277)
RUV_plasma	0.679	0.108	—	—	0.664 (0.489, 0.900)
RUV_CSF	1.19	0.125	—	—	1.15 (0.941, 1.391)
Lambda_plasma	0.280	0.107	—	—	0.275 (0.064, 0.482)
Lambda_CSF	0.285	0.107	—	—	0.279 (0.066, 0.485)
Delta_plasma	−0.174	0.052	—	—	−0.178 (−0.287, −0.063)

SE, standard error from NONMEM covariance step; CV, coefficient of variation; IIV, between-subject variability; Cov, covariance; RUV, residual error; θ, estimated covariate effect.

Lambda and delta are parameters from the dynamic-transform-both-side approach for residual error modelling (more specifically, lambda is the shape parameter and delta is the scedasticity parameter; together they are a part of the Box–Cox power parameter, zeta = lambda + delta).

a Indicates that the value is on the logit scale.

**Figure 2.**
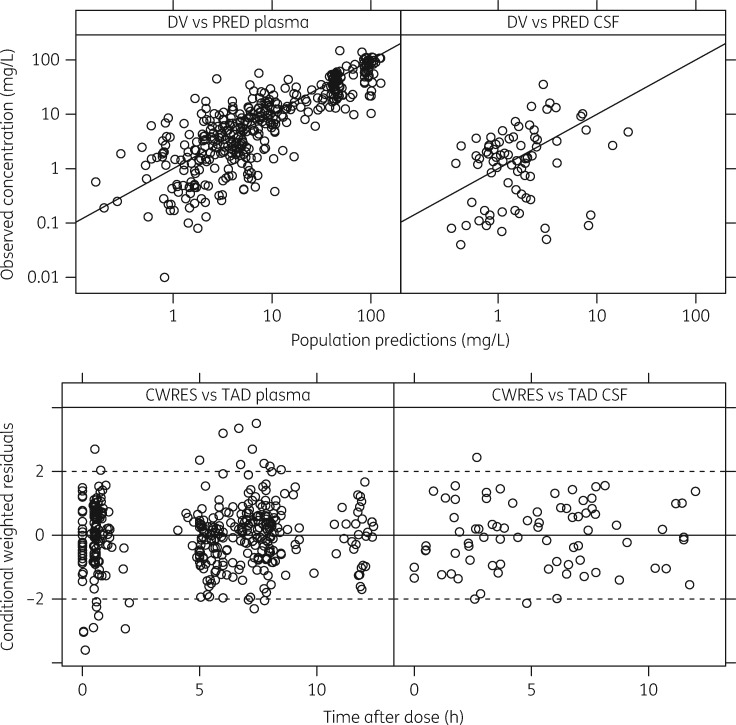
Basic goodness-of-fit plots for the final model. The top two plots show observed concentration (DV) versus population predictions (PRED) for plasma and CSF samples. The bottom two plots show CWRES versus time after dose (TAD) for plasma and CSF samples.

**Figure 3.**
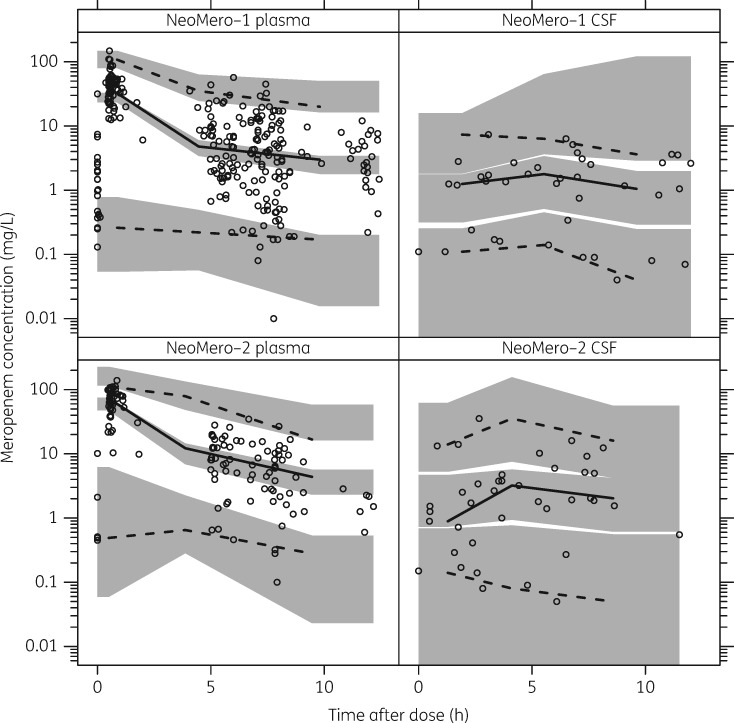
Visual predictive check showing the 2.5th, 50th and 97.5th percentiles of the observed data (lines and open circles) compared with the 95% CIs of the corresponding simulations from the final model (shaded areas). The top panel shows plasma and CSF for NeoMero-1 and the bottom panel shows plasma and CSF for NeoMero-2.

The diagnostic plots showed adequate fit to the data (i.e. agreement between the measured and predicted concentrations was observed and there was no particular trend in the residual plots) (Figure 2) and the visual predictive check (using 1000 replicates) confirmed that the model had good simulation properties (Figure 3).

### In vitro PD target reached in all culture-proven cases

**Figure 4.**
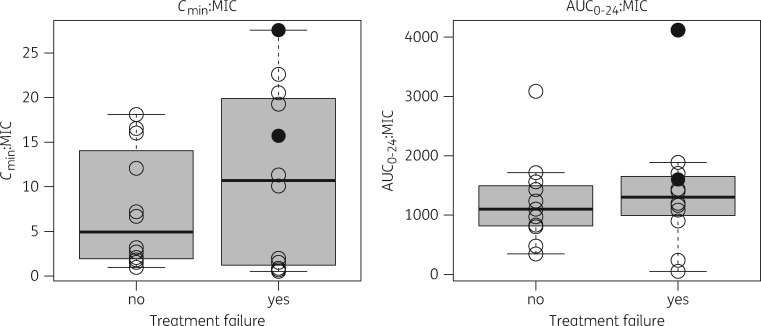
Box-and-whisker plots of the probability of treatment failure versus *C*_min_:MIC (left) and AUC_0–24_:MIC (right) ratios for the LOS patients with Gram-negative organisms and corresponding meropenem MIC. Open circles represent the raw data for each patient and filled circles represent patients who died.

**Figure 5.**
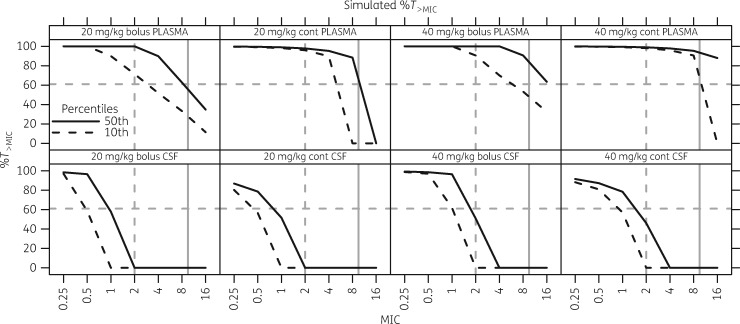
Simulated %*T*_>MIC_ for various dose schemes. The top row gives values for plasma and the bottom row gives values for CSF. A comparison of 20 mg/kg versus 40 mg/kg as either a bolus or continuous infusion (cont) is shown. The continuous black line gives the %*T*_>MIC_ for the typical patient (50th percentile), whereas the broken black line gives the %*T*_>MIC_ for at least 90% of patients (10th percentile). Targets are highlighted by grey lines: the horizontal broken grey line represents 61%*T*_>MIC_, the vertical broken grey line represents an MIC cut-off of 2 mg/L and the continuous grey line represents 10 mg/L (5 × MIC).

In the NeoMero-1 study there were 24 individuals with culture-proven LOS with a Gram-negative organism for which MIC values were available and at least 24 h of meropenem had been administered. Of these, 12 patients were considered to have been successfully treated (no need to modify the treatment course), whereas 12 patients failed (2 died, 9 required treatment modification at the discretion of the treating physician and 1 still had unresolved symptoms). The mean MIC in the 12 successes was 0.27 mg/L, whereas in the 12 failures the mean MIC was 0.98 mg/L (*P *=* *0.38). All patients had a %*T*_>MIC_ above 61% (the proposed *in vitro*-derived target^8^); there was no difference in AUC_0–24_:MIC ratio (*P *=* *0.53) or in *C*_min_:MIC ratio (*P *=* *0.73) in patients classified as treatment failures versus successes (Figure 4). Simulations of %*T*_>MIC_ showed little difference between bolus and 30 min infusions (data not shown) and so a comparison of 20 and 40 mg/kg bolus versus continuous infusion are shown in Figure 5 (with a frequency of 8 hourly or 12 hourly for those <32 weeks gestation and <2 weeks PNA as per our study dosing).

## Discussion

This population PK model describing plasma and CSF disposition of meropenem in infants aged <90 days with LOS and/or bacterial meningitis represents the largest study of meropenem PK in infants aged ≤90 days to have been reported to date, which also included the highest number of collected CSF samples in this population. The generally accepted target of 40%*T*_>MIC_ is believed to be adequate for bactericidal effects of carbapenems,^5^ but a recent *in vitro* study has suggested that differences in the PK profile in neonatal patients means a higher target of 61%*T*_>MIC_ is warranted.^8^ All of our patients with culture-proven Gram-negative LOS achieved this target, but it should be noted that most MIC values were ≤0.25 mg/L. Simulations showed that 90% of patients should achieve 61%*T*_>MIC_ for organisms with an MIC of ≤2 mg/L and so our major finding is that, for meropenem-susceptible organisms, a 20 mg/kg bolus appears to be a sufficient dose for LOS (Figure 5).

A recent randomized controlled trial of continuous versus 30 min meropenem infusion in neonates with culture-proven infection found decreased mortality and ventilator support required in the continuous infusion group.^34^ Whilst no MICs were recorded in this study, which also found surprisingly high failure of microbial eradication at 7 days (30% overall), it is not the only clinical study to report improved outcomes with differing meropenem *C*_min_. High %*T*_>MIC_ has also been reported to be associated with improved clinical outcomes in adult lower respiratory tract infection,^9^ with a breakpoint of *C*_min_ of 5 times the MIC being associated with maximum benefit.

The simulations in Figure 5 show that the same dose given as a continuous infusion achieves higher *C*_min_:MIC ratios. Simulations from our model based on a breakpoint of 2 mg/L showed that standard 20 mg/kg dosing would achieve the *in vitro*-derived target of 61%*T*_>MIC_^8^ in 90% of patients, but if the MIC were to increase to 4 mg/L as organisms become intermediately resistant, it would be necessary to increase dosing to 40 mg/kg. The use of a continuous infusion with an 8 hourly dose of 40 mg/kg (i.e. 120 mg/kg in 24 h) would be required to achieve a *C*_min_:MIC ratio of >5 for an MIC of around 1 mg/L and continuous infusions do clearly increase plasma %*T*_>MIC_ (Figure 5). Simply moving to continuous infusions may, however, not be appropriate, particularly in this clinical setting where patients with LOS can go on to develop meningitis. As can be seen in Figure 5, continuous infusions give substantially lower CNS concentrations for the same total daily dose. This is likely due to low *C*_max_ resulting in lower peripheral concentrations. The association between longer meropenem infusions and lower *C*_max_ has also been previously shown.^14^

The data in our study have substantially increased the literature on meropenem CSF PK, which enables simulated dosing schemes to balance circulating and CSF concentrations. The only study that focused on meropenem plasma and CSF disposition in infants (<3 months of age) to date involved six patients, who provided nine CSF samples.^15^ Smith *et al.*^15^ reported that uptake of meropenem into the CSF was 70%, determined by comparing plasma and CSF concentrations at the same timepoint. This method is suboptimal since the CSF and plasma time courses vary as β-lactams enter the CSF through paracellular pathways,^35^ resulting in a delayed peak CSF concentration. Our model-based typical estimate for the fraction of meropenem penetration from plasma into the CSF was 8.4%, which is at the lower end of the values previously reported in the literature: 10% up to 30%^35^^,^^36^ or 40%^37^ and this could be because the meninges were not inflamed^13^ in many of the NeoMero-1 patients without meningitis; median CSF protein and CSF lactate concentrations were almost in normal ranges (1.2 g/L and 1.8 mmol/L, respectively). We did find a significant increase in CNS penetration with increasing CSF protein concentration, with penetration reaching over 40% when CSF protein concentration exceeded 6 g/L. Overall the CNS penetration results show that the fraction entering the CNS is low and comparable with other populations, although when inflammation is present, as evidenced by the presence of proteins in the CSF, penetration significantly increases.

The values of the PK parameters for a typical infant from this study (weight = 2.1 kg, postmenstrual age = 37.4 weeks, serum creatinine = 32 μmol/L, CSF protein concentration = 1.2 mmol/L and serum creatinine, standardized by postmenstrual age = 60 μmol/L) were: CL = 0.39 L/h and *V* = 1.17 L. These values are in agreement with what has been previously reported in the literature. For example, van den Anker *et al.*^16^ found that CL was 0.43 L/h and *V* was 0.97 L for a population of premature and mature infants. When only premature infants with an approximate weight of 1 kg were studied, the CL was lower, specifically 0.06 L/h^14^ and 0.15 L/h.^17^ A lower clearance of 0.13 L/h was also reported by Smith *et al.*;^15^ however, in all these cases the infants weighed around 1 kg, which would explain the lower CL estimate. This also is the reason for a 12-hourly frequency to be retained in the youngest premature age group.

Since meropenem showed low potential for nephrotoxicity,^10^^,^^38^ higher doses do not necessarily mean increased toxicity.^35^ Therefore, if needed, the doses could be increased or meropenem could be given more frequently.

### Conclusions

A PK model describing plasma and CSF meropenem data in young infants with confirmed or suspected LOS and/or meningitis was developed using data from one of the largest infant sepsis trials to have been conducted in this population. Dosing of 20 mg/kg as an 8 hourly bolus may be adequate for LOS at current MIC targets, but in future 40 mg/kg may be necessary owing to increasing pathogen MICs. Increasing infusion times (up to continuous infusion) improves circulating %*T*_>MIC_, but decreases CSF %*T*_>MIC_.
